# The association between insulin resistance indices and the occurrence of major adverse cardiovascular events in patients with premature myocardial infarction: a prospective cohort study

**DOI:** 10.3389/fnut.2026.1724362

**Published:** 2026-02-19

**Authors:** Yu Zhou, Yuhang Wang, Jingxi Chen, Lai Jiang, Ran Chu, Weiwei Tian, Jiaxin Wang, Yin Liu, Jing Gao

**Affiliations:** 1 Chest Hospital, Tianjin University, Tianjin, China; 2 Clinical School of Thoracic, Tianjin Medical University, Tianjin, China; 3 Department of Cardiology, Tianjin Chest Hospital, Tianjin, China; 4 Cardiovascular Institute, Tianjin Chest Hospital, Tianjin, China; 5 Tianjin Key Laboratory of Cardiovascular Emergency and Critical Care, Tianjin, China

**Keywords:** diabetes, insulin resistance, major adverse cardiovascular events, premature myocardial infarction, triglyceride glucose-body mass index

## Abstract

**Background:**

Insulin resistance (IR) alternative markers, including the triglyceride-glucose index (TyG), TyG combined with body mass index (TyG-BMI), and the triglyceride/high-density lipoprotein cholesterol ratio (TG/HDL-C), have been shown to be significantly associated with prognosis of acute myocardial infarction (AMI). However, the prognostic value of these markers in patients with premature myocardial infarction (PMI) remains unclear. This study aims to investigate the association between IR markers and major adverse cardiovascular events (MACEs) in PMI patients.

**Methods:**

This was a prospective cohort study that consecutively enrolled 1,688 PMI patients (male ≤ 50 years, female ≤ 55 years) from Tianjin Chest Hospital between February 2015 and December 2024. TyG, TyG-BMI and TG/HDL-C indices were calculated. The median follow-up time was 17.4 months (IQR: 11.4–31.9), with the endpoint being MACEs. IR indices were grouped by quartiles. The risk association between IR indices and MACEs was analyzed using Cox proportional hazards models and restricted cubic spline analysis. The predictive performance of IR indices was assessed using Harrell’s C-index, net reclassification improvement (NRI), integrated discrimination improvement (IDI).

**Results:**

Among 1,688 patients, 211 (12.5%) occurred MACEs. Restricted cubic spline analysis showed a positive nonlinear relationship between TyG-BMI and MACEs risk, while TyG and TG/HDL-C were linearly associated with MACEs risk. In the fully adjusted Cox proportional hazards model, the hazard ratios of occurring MACEs in the fourth quartile versus the first quartile were 2.88 [95% confidence interval (CI): 1.83–4.53] for TyG-BMI, 1.77 (95% CI: 1.11–2.82) for TyG, 1.44 (95% CI: 0.93–2.22) for TG/HDL-C. In the fourth quartile versus the first quartile of TyG-BMI, the hazard ratios of occurring MACEs were 3.85 (95% CI: 1.79–8.27) in patients with diabetes and 3.38 (95% CI: 1.78–6.43) in patients with high high-sensitivity C-reactive protein (hsCRP). Additionally, TyG-BMI demonstrated higher C-index, NRI and IDI for predicting MACEs risk in PMI patients.

**Conclusion:**

In patients with PMI, TyG-BMI is an independent predictor of MACEs demonstrating significantly superior predictive performance compared to TyG and TG/HDL-C. The association between elevated TyG-BMI and MACEs risk was significant in patients with diabetes and high hsCRP levels. The effect was particularly stronger in patients with diabetes.

## 1 Introduction

In recent years, the age of onset of acute myocardial infarction (AMI) tends to be younger (1). Patients with premature myocardial infarction (PMI) not only account for a significant proportion (PMI deaths account for approximately 12.5% of AMI deaths) (2) but also differ significantly from older patients in terms of etiology, risk factor profiles, and clinical prognosis (3–5). Given the rising prevalence of metabolic diseases and the projected increase in PMI burden, there is an urgent need to improve early risk identification and prognostic management strategies to prevent major adverse cardiovascular events (MACEs) in this special population.

Insulin resistance (IR) is considered to be a reduced sensitivity or responsiveness to the metabolic actions of insulin, including insulin-mediated glucose processing. Several studies have found that IR influences the onset and progression of type 2 diabetes and cardiovascular disease (6). The hyperinsulinemic-euglycemic clamp test is considered the gold standard for assessing IR, but it is not suitable for widespread clinical application due to its high cost and invasiveness (7). Therefore, it is crucial to establish practical and effective alternative indicators of IR. Currently, the triglyceride glucose index (TyG) and triglyceride/high-density lipoprotein cholesterol ratio (TG/HDL-C) have been widely accepted as reliable indices for the assessment of IR (8, 9). The TG/HDL-C index responds to lipid metabolism and is a reliable indicator for the assessment of IR and residual cardiovascular risk, with a significantly higher clinical value than a single lipid parameter (10). The TyG index, combined with triglyceride and fasting glucose indices, reflects the status of glucose-lipid metabolism and is strongly associated with mortality from coronary artery disease, ischemic stroke, and heart failure, as well as the risk of developing type 2 diabetes mellitus (11–13). Notably, IR has a bidirectional promotional relationship with obesity, obesity exacerbates IR through inflammation and endoplasmic reticulum stress, while IR further promotes metabolic dysregulation (14, 15). As a convenient indicator of obesity, body mass index (BMI) can independently increase the risk of cardiovascular disease and stroke (16–18) and synergistically amplify cardiovascular injury with IR. Based on this, the triglyceride-glucose body mass index (TyG-BMI), which combines the TyG index with the obesity index BMI, has become a reliable indicator of IR and cardiovascular risk, with several studies showing a strong association and predictive ability for cardiovascular disease (19, 20). However, research evidence on the association and predictive utility of IR alternative indicators such as TyG-BMI with the risk of developing MACEs in PMI groups with unique clinical characteristics is limited. In particular, direct comparative studies between TyG-BMI and other commonly used IR metrics (TyG, TG/HDL-C) in PMI patients are lacking, and it is unclear whether different glucose metabolic statuses (normal blood glucose, prediabetes mellitus, and diabetes mellitus) and different levels of high-sensitivity C-reactive protein (hsCRP) affect the predictive efficacy of these metrics.

Therefore, this study aims to assess the association between the TyG-BMI and the incidence of MACEs in PMI patients as well as to compare the predictive efficacy of TyG-BMI with TyG and TG/HDL-C indices in predicting the risk of MACEs in PMI patients. Additionally, we explore the association between TyG-BMI and MACEs occurrence in PMI patients across different glucose status subgroups and different levels of hsCRP, and we comprehensively evaluate the predictive value of IR indices for the risk of MACEs in PMI patients.

## 2 Materials and methods

### 2.1 Study design and study population

This study was a prospective cohort study that consecutively enrolled patients with acute myocardial infarction who underwent coronary angiography at Tianjin Chest Hospital between February 2015 and December 2024. The age requirements were ≤ 50 years for men and ≤ 55 years for women. The diagnostic criteria for acute myocardial infarction were indicated by elevated troponin levels (at least one measurement exceeding the 99th percentile of the reference range) and any of the following: symptoms of myocardial ischemia, new ischemic electrocardiographic changes, new evidence of viable myocardial loss or segmental ventricular wall motion abnormalities on imaging, and coronary angiography-confirmed coronary artery thrombosis. Exclusion criteria included any of the following: moderate to severe valvular heart disease, pulmonary embolism, severe inflammatory disease, malignant tumors, patients who did not undergo coronary angiography, and patients with incomplete clinical data.

**FIGURE 1:**
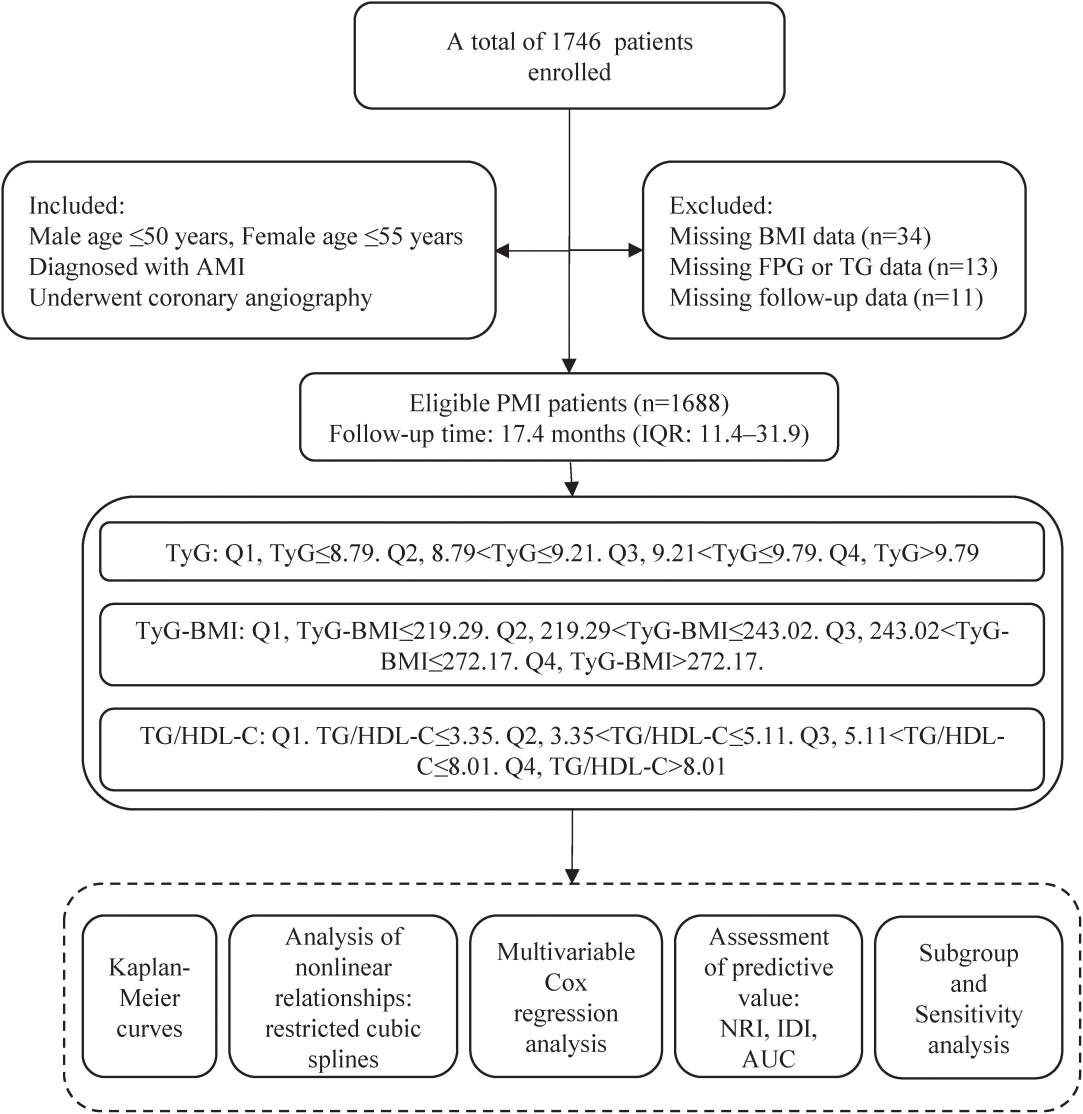
Flowchart of the study.

After excluding patients with incomplete clinical data and failed follow-up, a total of 1,688 PMI patients were included in this study. The study process was shown in Figure 1. Patients were grouped based on the quartiles of TyG, TyG-BMI, and TG/HDL-C levels at admission. This study was approved by the local research ethics committee and strictly adhered to the Declaration of Helsinki.

### 2.2 Data collection

Clinical data was collected by trained clinicians from medical electronic health record systems. The data collected included demographic information (age, gender), physical measurement data (height, weight, systolic blood pressure (SBP) and diastolic blood pressure (DBP), heart rate), lifestyle data (smoking status, alcohol consumption status), past medical history (history of hypertension, diabetes, family history of coronary heart disease, previous myocardial infarction (MI), atrial fibrillation, hyperlipidemia, angina pectoris), laboratory data (fasting plasma glucose (FPG), hemoglobin A1c (HbA1c), total cholesterol (TC), triglycerides (TG), low-density lipoprotein cholesterol (LDL-C), high-density lipoprotein cholesterol (HDL-C), very-low-density lipoprotein cholesterol (VLDL-C), apolipoprotein B (ApoB), serum creatinine (Cr), serum uric acid (UA), high-sensitivity C-reactive protein, N-terminal pro-B-type natriuretic peptide (NT-proBNP)). Estimated glomerular filtration rate (eGFR) was calculated using the CKD-EPI 2021 formula (21). The imaging data included left ventricular ejection fraction (LVEF) and anterior-posterior diameter of the left atrium (LA). LVEF was measured using two-dimensional echocardiography via the modified two-plane Simpson method (22). BMI was defined as weight (kg)/height (m^2^). All patients underwent coronary angiography during hospitalization. Angiographic data included the number of diseased vessels. Information on medication therapy was also collected (aspirin, P2Y12 receptor antagonists, beta-blockers, angiotensin-converting enzyme inhibitors/angiotensin II receptor antagonists (ACEI/ARB), and statins). The percutaneous coronary intervention (PCI) strategy of this study is that primary PCI is only performed for the culprit lesions. For ST-segment elevation myocardial infarction (STEMI) patients with multi-vessel lesions, if ischemic symptoms still occur after primary PCI, elective non-culprit vessel revascularization treatment is considered.

Calculation methods for indicators:


T⁢y⁢G=ln⁢[T⁢G⁢(m⁢g/d⁢l)×F⁢P⁢G⁢(m⁢g/d⁢l)/2]

T⁢y⁢G-B⁢M⁢I=ln⁢[T⁢G⁢(m⁢g/d⁢l)×F⁢P⁢G⁢(m⁢g/d⁢l)/2]×B⁢M⁢I

T⁢G/H⁢D⁢L-C=T⁢G⁢(m⁢g/d⁢l)/H⁢D⁢L-C⁢(m⁢g/d⁢l)

Hypertension is defined as patients self-reporting hypertension, currently taking antihypertensive medication, or having a systolic blood pressure of ≥ 140 mmHg and a diastolic blood pressure of ≥ 90 mmHg (23). Diabetes is defined as patients self-reporting diabetes, currently using insulin or oral hypoglycemic agents, or having a FPG level ≥ 7.0 mmol/L and a HbA1c level ≥ 6.5%. Prediabetes is defined as an FPG level of 5.6–6.9 mmol/L and an HbA1c level of 5.7–6.4% (24).

### 2.3 Endpoints

Follow-up information was obtained through the following methods: face-to-face conversation with patients in the hospital, collection of outpatient medical records via the hospital administration system, and telephone contact with patients to inquire about their recovery progress.

Major adverse cardiovascular events, including cardiac death, rehospitalization for severe heart failure, nonfatal myocardial infarction, nonfatal stroke, rehospitalization for unstable angina, and target lesion revascularization defined as unplanned revascularization driven by an ischemic symptom or event, including percutaneous coronary intervention and coronary artery bypass grafting, are the primary endpoints of this study. Cardiac death was defined as death where the primary cause was attributable to a cardiovascular event, including deaths related to acute myocardial infarction, sudden cardiac death, death from heart failure, and other cardiovascular causes (25, 26).

### 2.4 Statistical analysis

This study summarized baseline characteristics based on the quartiles of the TyG-BMI index. For continuous variables following a normal distribution, descriptive statistics were presented as mean ± standard deviation, and between-group differences were inferred using analysis of variance (ANOVA). For continuous variables that did not follow a normal distribution, statistical descriptions were provided using the median and interquartile range, and the Kruskal-Wallis H test was used to assess between-group differences. Categorical variables were characterized by frequency and percentage, and the chi-square test was used to assess between-group differences. Multiple imputation was used to handle missing data, and the generalized variance inflation factor (GVIF) analysis was employed to identify variables with multicollinearity.

With MACEs incidence as the primary outcome event, Cox regression models were used to analyze the hazard ratio (HR) and 95% confidence interval (95% CI) of IR indices (grouped by quartiles) with MACEs incidence. Estimates were made for the crude model and three adjusted models. Model 1 was adjusted for age and gender, and Model 2 was adjusted for age, gender, and prior medical history (smoking and drinking status, hypertension, diabetes, hyperlipidemia, and history of myocardial infarction). Model 3 was adjusted for the factors in Model 2 plus other indicators including LVEF, LA, NT-proBNP, SBP, DBP, hsCRP, ApoB, UA, and eGFR. Restricted cubic spline (RCS) regression was used to explore potential nonlinear associations between IR indices and the incidence of MACEs.

Kaplan-Meier curves were used to illustrate the survival rates for MACEs grouped by IR indices quartile. The predictive value was evaluated using receiver operating characteristic (ROC) curves, and the area under the curve (AUC) was calculated to quantify the predictive ability of the IR indices for the occurrence of MACEs. Additionally, we calculated Harrell’s C-index, net reclassification index (NRI), and integrated discrimination improvement (IDI) to evaluate the incremental performance of the IR indices relative to traditional risk factors in predicting the incidence of MACEs in PMI patients.

We compared the incremental prognostic value of the IR indices with the Secondary Manifestations of ARTerial disease (SMART) risk score. The SMART risk score was calculated according to the methodology described by Hageman et al. (27), incorporating the following variables: age, gender, current smoking status, diabetes, systolic blood pressure (mmHg), non-HDL cholesterol (mmol/L), first atherosclerotic cardiovascular disease (ASCVD) manifestation as coronary artery disease (CAD), estimated glomerular filtration rate (eGFR) (mL/min/1.73 m^2^), hsCRP (mg/L), years since first clinical manifestation of ASCVD, and baseline use of aspirin or equivalent antithrombotic therapy (including other antiplatelet agents and oral anticoagulants).

To investigate the association between the IR indices and the incidence of MACEs in PMI patients with different glucose metabolism statuses and different levels of hsCRP, a subgroup analysis was conducted on this population, and subgroup and interaction analyses were performed for various age groups, genders, smoking and drinking statuses. Additionally, in the sensitivity analysis, the association between the IR indices and the risk of MACEs was evaluated by removing all missing data to ensure the robustness of the results. The association between the IR indices and the risk of single endpoints was also evaluated. The proportion of missing data for key variables is as follows: LVEF 3.67%, LA 3.85%, NT-proBNP 8.18%, hsCRP 4.32%, ApoB 4.86%, UA 0.59%, Cr 0.59%. All statistical analyses were conducted using R software (version 4.3.1), and a two-sided *P* < 0.05 was considered statistically significant.

## 3 Results

### 3.1 Baseline characteristics of study participants

This study included a total of 1,688 patients, with a median age of 42 years (IQR: 37–44) in the overall population. Males accounted for 88.6% of the study population, and there were no significant differences in gender and age distribution among the groups (*P* > 0.05). Baseline characteristics were shown in Table 1. Patients were grouped based on their TyG-BMI levels at admission, divided into quartiles (Q1-Q4, *n* = 422 per group). As TyG-BMI levels increased, BMI, SBP and DBP were significantly higher in the high TyG-BMI group (all *P* < 0.001). The prevalence of diabetes and hypertension was significantly higher in the Q4 group (*P* < 0.001). Additionally, they exhibited more severe lipid metabolism abnormalities, characterized by elevated TG and VLDL-C, while HDL-C levels decreased (all *P* < 0.001). Among inflammatory and glucose metabolism markers, the high TyG-BMI group showed significantly elevated levels of hsCRP, FPG, and HbA1c (all *P* < 0.001).

**TABLE 1:** Baseline characteristics of study population stratified by quartiles of TyG-BMI.

Characteristics	Quartile 1 ( ≤ 219.29) (*n* = 422)	Quartile 2 (219.29–243.02) (*n* = 422)	Quartile 3 (243.02–272.17) (*n* = 422)	Quartile 4 ( > 272.17) (*n* = 422)	*P*
Age, years	42.0 (38.0, 45.0)	42.0 (38.0, 44.0)	41.0 (38.0, 44.0)	40.0 (36.0, 44.0)	< 0.001
Male, n(%)	347 (82.2)	378 (89.6)	388 (91.9)	383 (90.8)	< 0.001
BMI, kg/m^2^	22.9 (21.5, 24.2)	25.6 (24.4, 26.3)	27.0 (25.9, 28.1)	30.9 (28.4, 33.0)	< 0.001
HRate, bpm	75.0 (66.0, 84.0)	75.0 (67.0, 85.0)	75.0 (68.0, 86.0)	78.0 (70.0, 89.8)	0.001
SBP, mmHg	130.0 (118.3, 140.0)	130.0 (119.0, 141.0)	132.0 (123.0, 145.0)	135.0 (123.0, 150.0)	< 0.001
DBP, mmHg	79.0 (70.0, 89.8)	79.0 (70.0, 90.0)	80.0 (74.0, 90.0)	81.5 (74.0, 95.0)	< 0.001
**Medical history, n(%)**					
Smoking	262 (62.1)	273 (64.7)	264 (62.6)	255 (60.4)	0.644
Drinking	140 (33.2)	148 (35.1)	130 (30.8)	131 (31.0)	0.509
Hypertension	182 (43.1)	183 (43.4)	218 (51.7)	227 (53.8)	0.001
Diabetes	63 (14.9)	67 (15.9)	96 (22.7)	115 (27.3)	< 0.001
Prior MI	12 (2.8)	28 (6.6)	25 (5.9)	21 (5.0)	0.069
Hyperlipidemia	71 (16.8)	89 (21.1)	115 (27.3)	85 (20.1)	0.003
Angina	56 (13.3)	60 (14.2)	54 (12.8)	35 (8.3)	0.041
Family CAD	38 (9.0)	45 (10.7)	44 (10.4)	27 (6.4)	0.119
AF	3 (0.7)	11 (2.6)	4 (0.9)	3 (0.7)	0.035
**Laboratory data**					
hsCRP, mg/L	4.0 (1.7, 9.0)	4.4 (1.9, 9.4)	5.2 (2.7, 9.3)	6.2 (3.2, 10.7)	< 0.001
Cr, μmol/L	74.0 (64.0, 84.0)	76.0 (65.0, 85.0)	73.8 (64.0, 84.0)	72.8 (63.0, 83.0)	0.129
eGFR, mL/min/1.73 m^2^	110.6 (100.4, 115.5)	110.9 (98.5, 116.5)	111.1 (101.0, 117.2)	113.3 (103.4, 118.8)	0.002
UA, μmol/L	342.5 (280.3, 398.8)	355.0 (287.0, 421.5)	371.0 (310.3, 439.0)	378.5 (313.3, 463.8)	< 0.001
ALT, U/L	40.0 (26.2, 60.0)	44.2 (27.6, 67.5)	43.0 (30.2, 68.5)	48.6 (32.3, 75.1)	< 0.001
AST, U/L	108.5 (48.4, 221.8)	109.8 (44.9, 214.2)	104.5 (44.1, 214.8)	125.0 (50.9, 229.3)	0.320
FPG, mmol/L	5.3 (4.7, 6.1)	5.5 (4.9, 6.8)	6.2 (5.2, 8.2)	7.1 (5.7, 10.2)	< 0.001
HbA1c, %	5.6 (5.3, 6.0)	5.7 (5.4, 6.3)	6.0 (5.5, 7.3)	6.5 (5.7, 8.6)	< 0.001
TC, mmol/L	4.5 (3.8, 5.3)	4.7 (4.0, 5.4)	4.9 (4.3, 5.6)	5.0 (4.4, 5.7)	< 0.001
TG, mmol/L	1.4 (1.1, 2.0)	1.8 (1.4, 2.5)	2.4 (1.8, 3.4)	3.1 (2.1, 4.8)	< 0.001
HDL-C, mmol/L	1.0 (0.9, 1.2)	0.9 (0.8, 1.1)	0.9 (0.8, 1.0)	0.9 (0.8, 1.0)	< 0.001
LDL-C, mmol/L	3.0 (2.4, 3.7)	3.2 (2.4, 3.7)	3.2 (2.6, 3.9)	3.1 (2.5, 3.8)	0.049
VLDL-C, mmol/L	0.4 (0.3, 0.6)	0.5 (0.3, 0.7)	0.6 (0.5, 1.0)	0.8 (0.5, 1.4)	< 0.001
ApoA1, g/L	1.1 (1.0, 1.2)	1.1 (1.0, 1.3)	1.1 (1.0, 1.3)	1.2 (1.0, 1.3)	0.002
ApoB, g/L	1.0 (0.8, 1.2)	1.1 (0.9, 1.3)	1.2 (1.0, 1.4)	1.3 (1.1, 1.5)	< 0.001
NT-proBNP, pg/mL	226.9 (66.5, 739.5)	186.2 (51.4, 554.2)	146.0 (31.6, 448.5)	83.6 (6.2, 315.4)	< 0.001
**Echocardiography**					
LA, mm	35.0 (33.0, 38.0)	36.0 (34.0, 38.0)	36.0 (34.0, 39.0)	37.0 (35.0, 40.0)	< 0.001
LV, mm	51.0 (48.0, 53.8)	51.0 (49.0, 54.0)	52.0 (49.0, 55.0)	52.0 (49.0, 55.0)	< 0.001
LVEF, %	52.0 (46.0, 57.0)	52.0 (46.0, 57.0)	53.0 (47.0, 56.0)	51.0 (46.0, 56.0)	0.118
SMART risk score, %	72.0 (61.0, 84.0)	76.0 (64.0, 86.0)	81.0 (69.0, 89.0)	84.0 (72.0, 92.0)	< 0.001
**Type of AMI**					
STEMI	314 (74.4)	340 (80.6)	336 (79.6)	343 (81.3)	0.060
**Number of diseased vessels, %**					
No obvious lesion	18 (4.3)	6 (1.4)	10 (2.4)	11 (2.6)	0.152
1-vessel disease	174 (41.2)	172 (40.8)	157 (37.2)	145 (34.4)	
2-vessel disease	108 (25.6)	114 (27.0)	124 (29.4)	129 (30.6)	
3-vessel disease	122 (28.9)	130 (30.8)	131 (31.0)	137 (32.5)	
**Number of stents, %**					
0	96 (22.7)	94 (22.3)	94 (22.3)	90 (21.3)	0.760
1	244 (57.8)	232 (55.0)	233 (55.2)	248 (58.8)	
2	67 (15.9)	69 (16.4)	75 (17.8)	62 (14.7)	
≥ 3	15 (3.6)	27 (6.4)	20 (4.7)	22 (5.2)	
**Treatment**					
Aspirin, %	420 (99.5)	420 (99.5)	422 (100.0)	421 (99.8)	0.531
Statin, %	419 (99.3)	413 (97.9)	412 (97.6)	410 (97.2)	0.145
P2Y12 receptor antagonist, %	421 (99.8)	420 (99.5)	421 (99.8)	422 (100.0)	0.571
Beta blocker, %	327 (77.5)	322 (76.3)	337 (79.9)	335 (79.4)	0.563
ACEI/ARB, %	266 (63.0)	259 (61.4)	299 (70.9)	313 (74.2)	< 0.001
**Outcome, n(%)**					
MACEs	35	49 (11.6)	63 (14.9)	64 (15.2)	0.007
Cardiac death	4 (0.9)	5 (1.2)	5 (1.2)	5 (1.2)	0.984
Unstable angina	7 (1.7)	9 (2.1)	18 (4.3)	19 (4.5)	0.032
Non-fatal Stroke	0 (0.0)	2 (0.5)	2 (0.5)	3 (0.7)	0.436
Non-fatal MI	5 (1.2)	5 (1.2)	7 (1.7)	5 (1.2)	0.907
HF	9 (2.1)	10 (2.4)	8 (1.9)	9 (2.1)	0.973
Revascularization	10 (2.4)	18 (4.3)	23 (5.5)	23 (5.5)	0.094

Data are described as median (interquartile range) for continue variables and n (%) for categorical variables. MI, myocardial infarction; CAD, coronary artery disease; STEMI, ST-segment elevation myocardial infarction; NSTEMI, non-ST-segment elevation myocardial infarction; HRate, heart rate; hsCRP, high-sensitivity c-reactive protein; HbA1c, glycosylated hemoglobin; FPG, fasting plasma glucose; TC, total cholesterol; TG, triglycerides; HDL-C, high-density lipoprotein cholesterol; LDL-C, low-density lipoprotein cholesterol; VLDL-C, very-low-density lipoprotein cholesterol; ApoA1, apolipoprotein A1; ApoB, apolipoprotein B; TyG-BMI, triglyceride glucose body mass index; Cr, creatinine; UA, uric acid; ALT, alanine aminotransferase; AST, aspartate aminotransferase; NT-proBNP, N-terminal pro-B-type natriuretic peptide. Unstable angina, rehospitalization for unstable angina. HF, rehospitalization for severe heart failure.

#### 3.2 Association of IR indices with MACEs in participants with PMI

The median follow-up duration in this study was 17.4 months (IQR: 11.4–31.9). A total of 211 patients (12.5%) experienced at least one major adverse cardiovascular event (MACE) during follow-up. Compared with participants without MACE, those who experienced MACEs during follow-up had higher BMI, FPG, TC, TG, and LDL-C, as well as a higher rate of triple-vessel disease (38.9% in the MACE group vs. 29.7% in the non-MACE group) (Supplementary Table S1).

**FIGURE 2:**
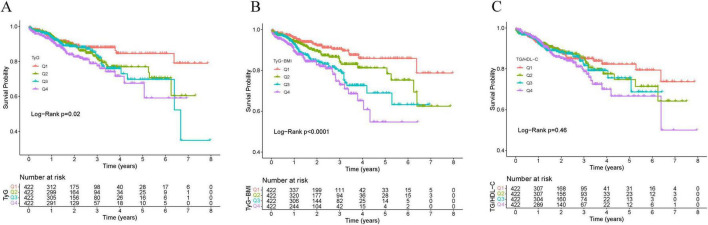
Kaplan–Meier analysis of IR indices with MACEs. **(A)** TyG. **(B)** TyG–BMI. **(C)** TG/HDL–C.

The incidence of MACEs progressively increased with rising TyG-BMI levels (*P* = 0.007). Kaplan-Meier curves describing survival rates by TyG-BMI quartiles showed a gradual decrease in patient survival with progressively higher TyG-BMI levels (*P* < 0.0001). A similar effect was observed across TyG quartiles, though less pronounced than the TyG-BMI results (*P* = 0.02). A comparable trend was noted across TG/HDL-C quartiles (*P* = 0.46) (Figure 2). After full adjustment for covariates, Cox regression analysis showed that, for each standard deviation increase in IR indices, the hazard ratios of occurring MACEs were 1.39 (95% CI: 1.22–1.60) for TyG-BMI, 1.30 (95% CI: 1.11–1.52) for TyG, and 1.15 (95% CI: 1.02–1.29) for TG/HDL-C. Compared with the first quartile, the hazard ratios of occurring MACEs in the fourth quartile were 2.88 (95% CI: 1.83–4.53) for TyG-BMI, 1.77 (95% CI: 1.11–2.82) for TyG, and 1.44 (95% CI: 0.93–2.22) for TG/HDL-C (Table 2). Multicollinearity analysis revealed no significant collinearity among variables (Supplementary Table 2). Univariate analysis of adjusted covariates was shown in Supplementary Figure 1.

**TABLE 2:** Associations of IR indices with incidences of MACEs in participants with PMI.

Case, n(%)		Crude		Model1		Model2		Model3	
		HR (95%CI)	*P*	HR (95%CI)	*P*	HR (95%CI)	*P*	HR (95%CI)	*P*
**TyG**									
Q1 ( ≤ 8.79)	42 (10.0)	Reference	–	Reference	–	Reference	–	Reference	–
Q2 (8.79–9.21)	53 (12.6)	1.37 (0.91–2.05)	0.132	1.37 (0.91–2.05)	0.131	1.39 (0.92–2.09)	0.115	1.37 (0.90–2.08)	0.139
Q3 (9.21–9.79)	54 (12.8)	1.44 (0.96–2.16)	0.075	1.45 (0.97–2.17)	0.073	1.50 (0.99–2.29)	0.057	1.41 (0.91–2.19)	0.122
Q4 ( > 9.79)	62 (14.7)	1.86 (1.25–2.76)	0.002	1.86 (1.26–2.77)	0.002	1.96 (1.28–3.00)	0.002	1.77 (1.11–2.82)	0.016
P for trend			0.002		0.002		0.002		0.003
Per 1 SD increase	–	1.29 (1.14–1.47)	< 0.001	1.29 (1.14–1.47)	< 0.001	1.31 (1.14–1.51)	< 0.001	1.30 (1.11–1.52)	0.001
**TyG-BMI**									
Q1 ( ≤ 219.29)	35 (8.3)	Reference	–	Reference	–	Reference	–	Reference	–
Q2 (219.29–243.02)	49 (11.6)	1.52 (0.99–2.35)	0.058	1.54 (0.99–2.37)	0.053	1.48 (0.96–2.30)	0.076	1.65 (1.06–2.56)	0.028
Q3 (243.02–272.17)	63 (14.9)	2.22 (1.47–3.36)	< 0.001	2.25 (1.49–3.42)	< 0.001	2.20 (1.45–3.36)	< 0.001	2.32 (1.50–3.59)	< 0.001
Q4 ( > 272.17)	64 (15.2)	2.81 (1.85–4.26)	< 0.001	2.87 (1.88–4.36)	< 0.001	2.70 (1.76–4.12)	< 0.001	2.88 (1.83–4.53)	< 0.001
P for trend			< 0.001		<0.001		< 0.001		<0.001
Per 1 SD increase	–	1.42 (1.25–1.60)	< 0.001	1.42 (1.26–1.61)	< 0.001	1.39 (1.23–1.57)	< 0.001	1.39 (1.22–1.60)	< 0.001
**TG/HDL–C**									
Q1 ( ≤ 3.35)	50 (11.8)	Reference	–	Reference	–	Reference	–	Reference	–
Q2 (3.35–5.11)	51 (12.1)	1.08 (0.73–1.59)	0.716	1.08 (0.73–1.60)	0.688	1.05 (0.71–1.56)	0.798	1.05 (0.71–1.57)	0.794
Q3 (5.11–8.01)	53 (12.6)	1.19 (0.80–1.75)	0.390	1.20 (0.81–1.78)	0.359	1.21 (0.81–1.80)	0.353	1.24 (0.82–1.87)	0.317
Q4 ( > 8.01)	57 (13.5)	1.34 (0.92–1.96)	0.133	1.36 (0.92–2.00)	0.120	1.46 (0.97–2.19)	0.067	1.44 (0.93–2.22)	0.098
P for trend			0.113		0.104		0.047		0.042
Per 1 SD increase	–	1.14 (1.02–1.27)	0.017	1.14 (1.03–1.27)	0.015	1.15 (1.04–1.29)	0.009	1.15 (1.02–1.29)	0.021

Model 1, adjusted for age and gender; Model 2, adjusted for age, gender, smoking, drinking, medical history of hypertension, diabetes, hyperlipidemia, and Prior MI; Model 3, adjusted for variables included in Model 2 and LVEF, LA, NT–proBNP, SBP, DBP, hsCRP, ApoB, UA, and eGFR. TyG–BMI, triglyceride glucose–body mass index; HR, hazard ratio; CI, confidence interval; SD, standard deviation.

**FIGURE 3:**
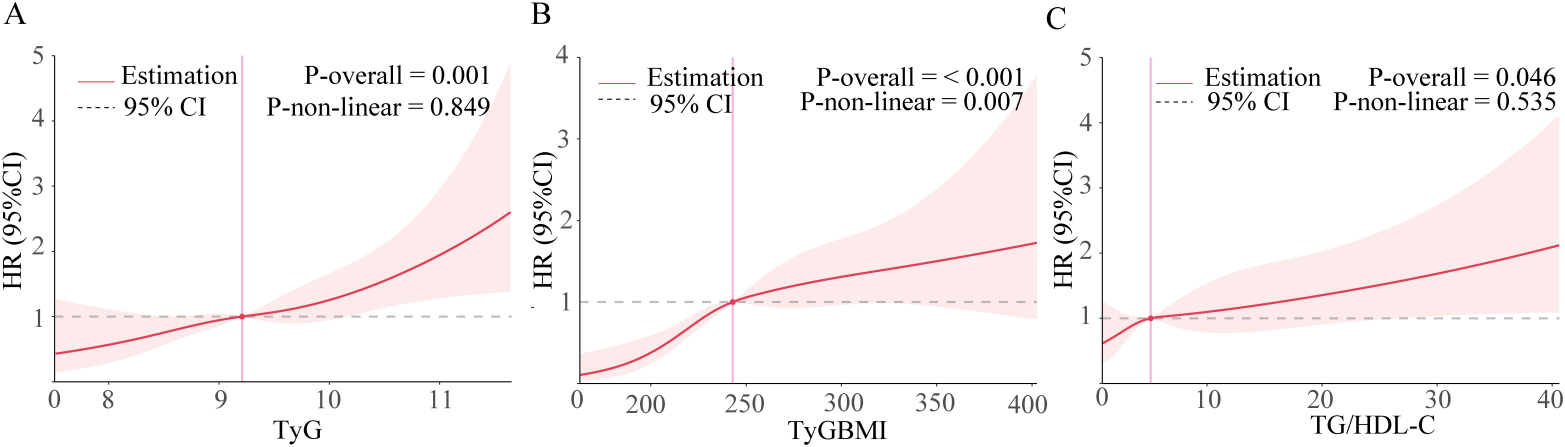
Nonlinear associations of IR indices with MACEs. The graph shows HRs for MACEs based on Cox hazards regression model 3 (adjusted for gender, age, smoking, drinking, and history of hypertension, diabetes, hyperlipidemia, Prior MI, and LVEF, LA, NT–proBNP, SBP, DBP, hsCRP, ApoB, UA, eGFR). Solid lines indicate HRs. Shadow shapes indicate 95% CIs. **(A)** TyG. **(B)** TyG–BMI. **(C)** TG/HDL–C.

RCS analysis depicted the dose-response relationship between IR indices and MACEs risk after full covariate adjustment (Figure 3). It indicated a nonlinear association between TyG-BMI and MACEs risk (*P* = 0.007), while both TyG and TG/HDL-C showed linear associations with MACEs risk (*P* = 0.849, *P* = 0.535). Given the nonlinear relationship between TyG-BMI and MACEs risk, we employed threshold effect analysis to further clarify the effect of TyG-BMI. When TyG-BMI was below the inflection point, the hazard ratio of occurring MACEs for each standard deviation increase was 3.11 (95% CI: 1.39–7.43). Above the inflection point, the hazard ratio was 1.22 (95% CI: 1.06–1.41) (Table 3).

**TABLE 3:** Threshold effect analysis of TyG–BMI with MACEs in participants with PMI.

	HR (95%CI)	*P*-value
**TyG–BMI (Per 1 SD increase)**		
Inflection point	211.95	–
Before inflection point	3.11 (1.39–7.43)	0.006
After inflection point	1.22 (1.06–1.41)	0.006
P for Log–likelihood ratio		0.002

Model was adjusted for gender, age, smoking, drinking, and history of hypertension, diabetes, hyperlipidemia, Prior MI, and LVEF, LA, NT–proBNP, SBP, DBP, hsCRP, ApoB, UA, and eGFR.

### 3.3 Association of IR indices with MACEs in participants with different glucose statuses and different levels of hsCRP

**FIGURE 4:**
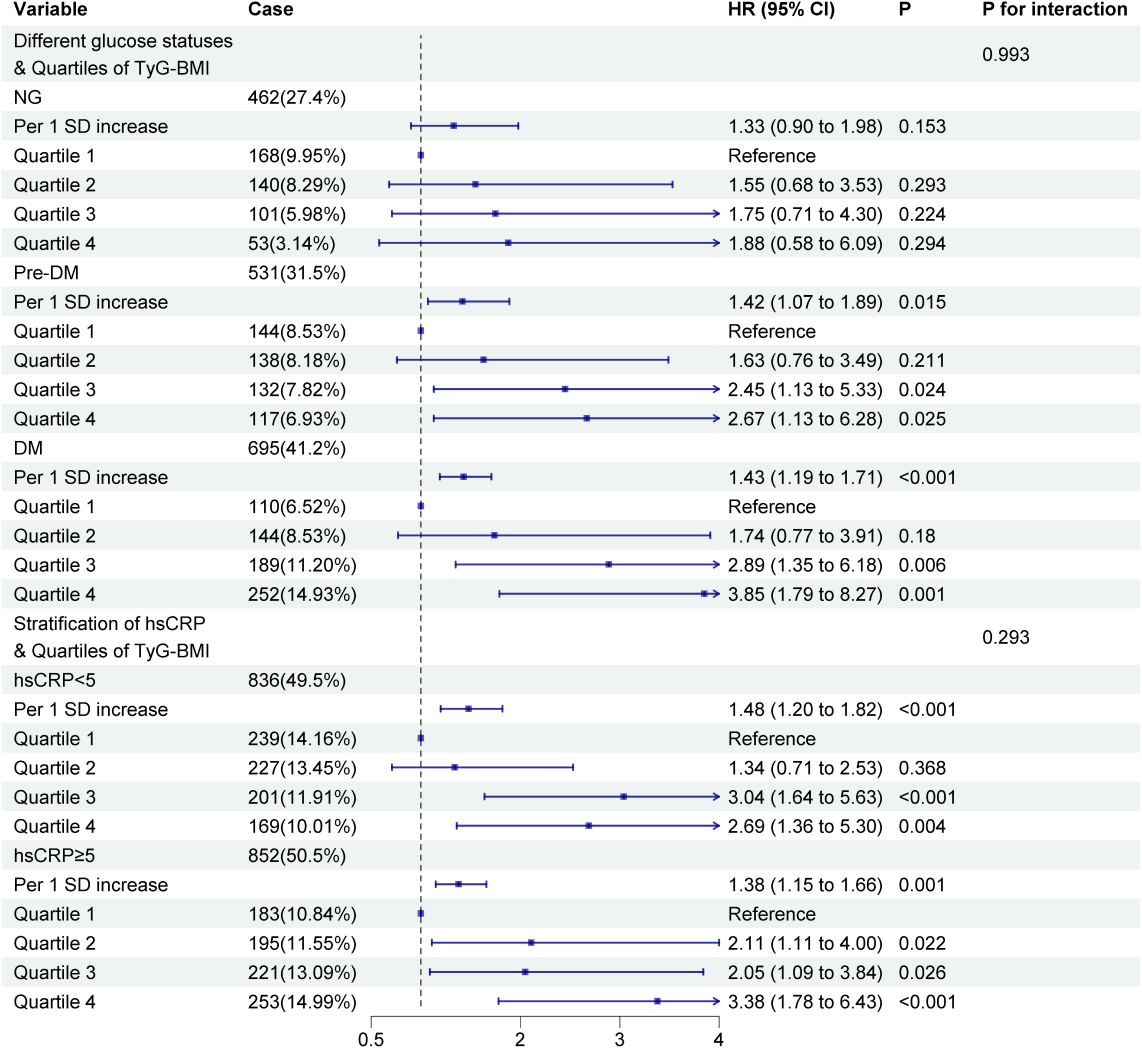
Association of TyG–BMI with MACEs in participants with different glucose statuses and different levels of hsCRP.

The above results indicated that TyG-BMI was the best risk indicator. Therefore, this study further investigated the association between TyG-BMI and the risk of MACEs occurring under different glucose metabolism statuses and different levels of hsCRP. As shown in Figure 4, in the prediabetic state, higher quartiles of TyG-BMI were significantly associated with MACEs risk (Q3, HR: 2.45, 95% CI: 1.13–5.33; Q4, HR: 2.67, 95% CI: 1.13–6.28). In patients with diabetes, higher quartiles of TyG-BMI showed significant associations with MACEs risk (Q3, HR: 2.89, 95% CI: 1.35–6.18; Q4, HR: 3.85, 95% CI: 1.79–8.27). In patients with high levels of hsCRP, higher quartiles of TyG-BMI were significantly associated with MACEs risk (Q3, HR: 2.05, 95% CI: 1.09–3.84; Q4, HR: 3.38, 95% CI: 1.78–6.43).

**FIGURE 5:**
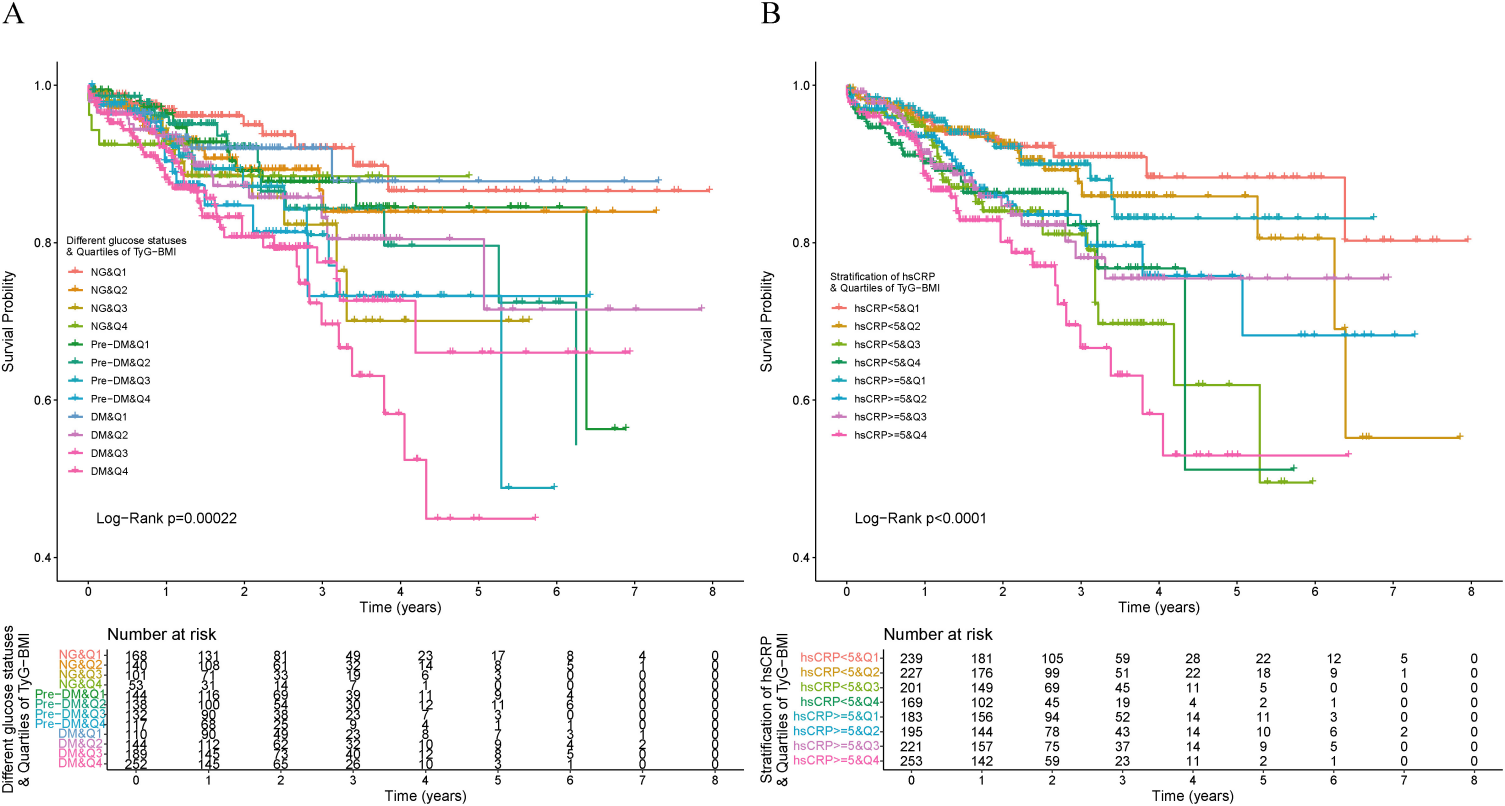
Kaplan–Meier analysis of TyG–BMI with MACEs in participants with different glucose statuses and different levels of hsCRP. **(A)** Participants with different glucose statuses. **(B)** Participants with different levels of hsCRP.

The Kaplan-Meier curves revealed that participants with the highest quartile of TyG-BMI and diabetes had the lowest survival rate (Figure 5A), followed by the participants with the highest quartile of TyG-BMI and those with high levels of hsCRP (Figure 5B).

### 3.4 Incremental predictive values of IR indices in participants with PMI

**FIGURE 6:**
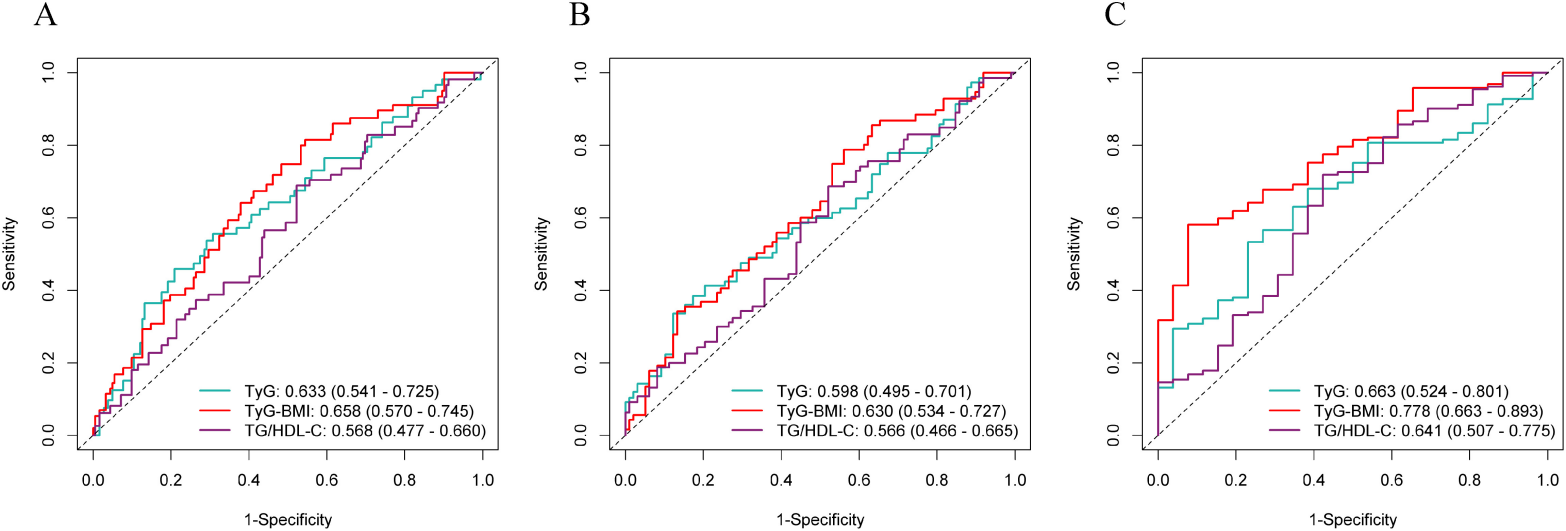
Receiver operating characteristic curves for IR indices predicting risk of MACEs. **(A)** 2–year ROC curve. **(B)** 3–year ROC curve. **(C)** 5–year ROC curve.

We further investigated whether incorporating the IR indices into the baseline model could improve predictive ability for MACEs risk in PMI patients. As shown in Table 4, compared with the baseline model (0.621, 95% CI: 0.544–0.699), the TyG-BMI model demonstrated a higher C-statistic (0.646, 95% CI: 0.572–0.720), a more significant NRI (0.184, 95% CI: 0.040–0.328), and IDI (0.0044, 95% CI: 0.0007–0.0081). The C-statistic of the TyG model was 0.635 (95% CI: 0.560–0.711), with statistical significance only in NRI (0.167, 95% CI: 0.023–0.311). The C-statistic for the TG/HDL-C model was 0.624 (95% CI: 0.547–0.702). Furthermore, when TyG-BMI was used as an independent predictor, the AUC values at 2, 3, and 5 years reached 0.658 (95% CI: 0.570–0.745), 0.630 (95% CI: 0.534–0.727), and 0.778 (95% CI: 0.663–0.893) (Figure 6).

**TABLE 4:** Additional predictive value of IR indices for predicting the risk of MACEs.

	C–Statistics (95%CI)	*P* value	NRI (95%CI)	*P* value	IDI (95%CI)	*P* value
Basic model	0.621 (0.544–0.699)	–	Reference	–	Reference	–
Basic model+ TyG	0.635 (0.560–0.711)	0.020	0.167 (0.023–0.311)	0.023	0.0027 (–0.0004–0.0058)	0.090
Basic model+ TyG–BMI	0.646 (0.572–0.720)	0.173	0.184 (0.040–0.328)	0.012	0.0044 (0.0007–0.0081)	0.020
Basic model+ TG/HDL–C	0.624 (0.547–0.702)	0.230	0.047 (–0.093–0.188)	0.508	0.0018 (–0.0012–0.0048)	0.236

The basic model included age, gender, smoking, drinking, medical history of hypertension, diabetes, hyperlipidemia, prior AMI, LVEF, LA, NT–proBNP, SBP, DBP, hsCRP, ApoB, UA, and eGFR.

We calculated the SMART risk score to predict the 5-year risk of MACEs occurrence and compared the incremental prognostic value of the IR indices based on this score (Table 5). Results showed that compared with the SMART risk score (0.611, 95% CI: 0.458–0.765), the addition of the IR indices improved the model’s predictive capability (TyG: 0.732, 95% CI: 0.599–0.864; TyG-BMI: 0.788, 95% CI: 0.680–0.896; TG/HDL-C: 0.739, 95% CI: 0.620–0.857), while the inclusion of TyG-BMI significantly enhanced the model’s ability to stratify risk (NRI: 0.192, 95%CI: 0.049–0.336; IDI: 0.005, 95%CI: 0.0014–0.0087).

**TABLE 5:** Additional predictive value of IR indices for predicting the risk of MACEs compared to SMART risk score.

	C–Statistics (95%CI)	*P-*value	NRI (95%CI)	*P*-value	IDI (95%CI)	*P*-value
SMART	0.611 (0.458–0.765)	–	Reference	–	Reference	–
SMART+ TyG	0.732 (0.599–0.864)	0.242	0.039 (–0.104–0.182)	0.590	0.0039 (0.0005–0.0074)	0.023
SMART+ TyG–BMI	0.788 (0.680–0.896)	0.065	0.192 (0.049–0.336)	0.009	0.005 (0.0014–0.0087)	0.007
SMART+ TG/HDL–C	0.739 (0.620–0.857)	0.197	–0.0068 (–0.141–0.127)	0.921	0.0015 (–0.001–0.0039)	0.245

The basic model included age, gender, smoking, drinking, medical history of hypertension, diabetes, hyperlipidemia, prior AMI, LVEF, LA, NT–proBNP, SBP, DBP, hsCRP, ApoB, UA, and eGFR.

### 3.5 Subgroup and sensitivity analysis

**FIGURE 7:**
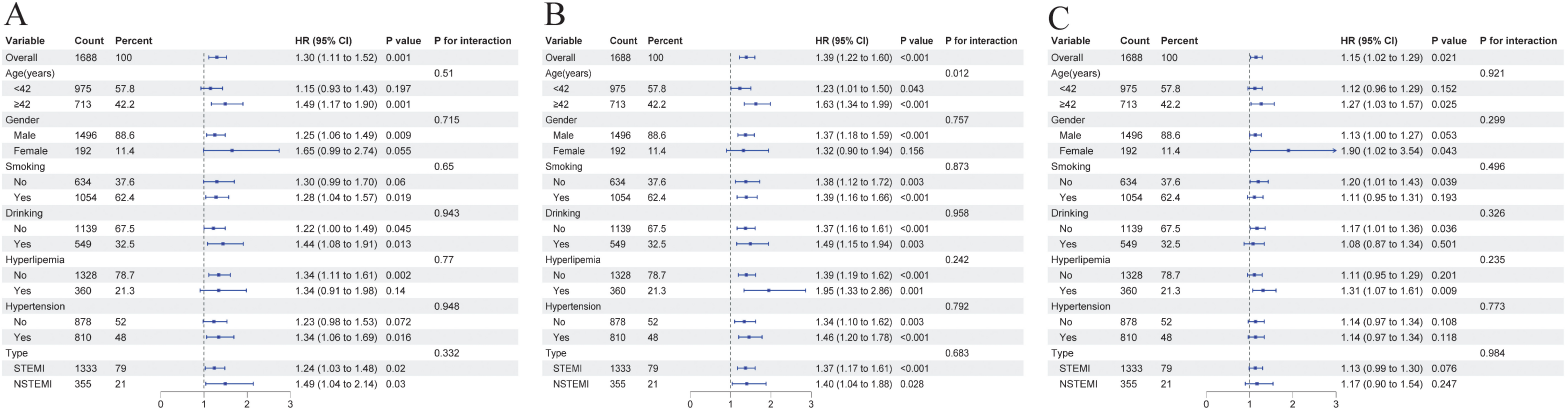
Subgroup analyses for the associations of IR indices with study outcomes in participants with PMI. **(A)** TyG. **(B)** TyG–BMI. **(C)** TG/HDL–C.

This study conducted subgroup analyses and sensitivity analyses to validate the robustness of the results. As shown in Figure 7, subgroup analyses revealed that the association between TyG-BMI and the risk of MACEs showed significant associations with age ( < 42 years vs. ≥ 42 years), smoking, alcohol consumption, hypertension, hyperlipidemia, or MI type. The TyG index was associated with MACEs risk in individuals aged ≥ 42 years, males, smokers, drinkers, those without hyperlipidemia, and those with hypertension, regardless of MI type, with no significant interactions. TG/HDL-C was associated with MACEs risk in non-drinkers and those with hyperlipidemia, showing no significant interactions with other subgroups. Sensitivity analysis after removing missing covariates from the fully adjusted model yielded similar results (Supplementary Figure 2). Sensitivity analysis for single endpoints demonstrated that TyG-BMI exhibits strong predictive capability for readmission due to unstable angina and target lesion revascularization (Supplementary Table 4).

## 4 Discussion

This study investigated the association between IR indices and the risk of MACEs in the PMI population, holding significant implications for prognosis management in this cohort. Key findings included: elevated TyG-BMI levels and elevated TyG levels were significantly associated with MACEs risk; elevated TyG-BMI levels demonstrated a stronger association and more predictive value with MACEs risk compared to TyG and TG/HDL-C; In the subgroup with diabetes and high levels of hsCRP, high quartiles of TyG-BMI were significantly associated with MACEs risk. TyG-BMI may serve as a prospective biomarker for predicting MACE risk in the PMI population.

In recent years, the TyG index has been widely recognized as a biomarker for metabolic and cardiovascular diseases (CVD) (28). Previous meta-analyses have consistently demonstrated that the TyG index is significantly associated with multiple cardiometabolic diseases. Specifically, elevated TyG index is associated with increased risk of heart failure (HR: 1.21, 95% CI: 1.14–1.29) (29), obstructive sleep apnea (standardized mean difference (SMD): 0.856, 95% CI: 0.579–1.132) (30), type 2 diabetes (HR: 1.72 for cardiovascular events) (31), peripheral arterial disease (SMD: 0.76, 95% CI: 0.65–0.88) (32), atrial fibrillation (SMD: 1.23, 95% CI: 0.71–1.75) (33). Furthermore, the risk of MACEs in the coronary artery disease cohort was 2.14 times higher in the highest TyG group compared to the lowest TyG group (HR: 2.14, 95% CI: 1.69–2.71) (34). Our study demonstrated that a high TyG index was significantly associated with MACEs, exhibiting this association in both NSTEMI and STEMI patients, consistent with previous research findings (35).

TyG-BMI serves as a novel composite indicator for assessing IR and cardiovascular risk, integrating the dual pathophysiological processes of dysglycemia and dyslipidemia (TyG index) with obesity burden (BMI). Currently, several studies confirm a significant association between TyG-BMI and cardiovascular morbidity as well as all-cause mortality. A large-scale study from the NHANES database found that individuals in the highest tertile of TyG-BMI had a 38% higher prevalence of CVD than those in the lowest tertile (36). According to Li et al.’s cohort study, among individuals with CKM syndrome stages 0–3, each 10-unit increase in TyG-BMI was associated with a 6.5% higher risk of developing CVD (37). Among patients with acute myocardial infarction undergoing PCI, the incidence of MACEs also increased with rising TyG-BMI levels (38). This study further validated the aforementioned association in the high-risk population of PMI, demonstrating a significant and strongly positive correlation between TyG-BMI and the risk of MACEs. Notably, the predictive value of TyG-BMI may exhibit population specificity. Another study found that elevated TyG-BMI scores were significantly associated with an increased incidence of MACEs among elderly or female patients undergoing PCI and drug-eluting stent (DES) implantation (39). In our study, TyG-BMI demonstrated significant predictive value for MACEs in male PMI patients, while no statistical significance was observed in females, likely due to sample size differences between genders. Furthermore, TG/HDL-C showed weaker associations with MACEs compared to TyG and TyG-BMI in both the general population and diabetic cohort. It could be because TG/HDL-C did not incorporate glucose and obesity information, focusing solely on dyslipidemia (HR: 1.15 in the general population). In contrast, TyG integrated dyslipidemia and dysglycemia (HR: 1.30 in the general population), while TyG-BMI further included the synergistic effect of obesity (HR: 1.39 in the general population). Therefore, TG/HDL-C failed to capture systemic IR information, limiting its utility in clinical risk assessment.

We evaluated the predictive capability of IR indices for MACEs incidence from three perspectives. First, we compared the predictive power of the baseline model with that of models incorporating IR indices. Results demonstrated that adding IR indices, particularly TyG and TyG-BMI, significantly enhanced the model’s ability to forecast risk. Second, we compared the independent predictive power of three IR indices (TyG, TyG-BMI, TG/HDL-C) at 2-, 3-, and 5-year time points. Results showed that TyG-BMI demonstrated the strongest predictive capability at all-time points. This may be attributable to the inclusion of the BMI component. A study evaluating the impact of BMI across the childhood-to-adulthood lifespan on CVD risk factors indicated that a persistent overweight trajectory from childhood to adulthood was associated with increased risks of hypertension and type 2 diabetes (40). Another study based on a Chinese cohort of 360,000 individuals demonstrated a significant dose-response relationship between early adulthood BMI and CVD risk. Compared to individuals with a BMI of 20.5–22.4 kg/m^2^, those with an early adulthood BMI exceeding 28 kg/m^2^ exhibited a 39% increased risk of CVD, while those with an early adulthood BMI exceeding 30 kg/m^2^ showed a 58% increased risk (41). Overall, elevated BMI increases the risk of CVD through multiple pathways, including inducing dyslipidemia, hypertension, IR, endothelial dysfunction, and structural cardiac changes (42, 43). Additionally, the SMART risk score was widely used to assess MACEs risk in ASCVD patients such as those with CAD. This study demonstrated that incorporating the IR indices provided incremental prognostic value, strongly supporting the IR indices’ contribution to risk stratification in PMI patients.

In this study, we conducted stratified analyses based on different glucose metabolism statuses, demonstrating the association between TyG-BMI and MACEs risk across varying glucose metabolism statuses. Previous studies have also focused on the association between IR and mortality in diabetic populations. For example, findings from a study using NHANES (2007–2016) data indicated that CVD prevalence increased with rising TyG among older diabetic adults in the United States, with a 48% higher CVD risk observed in the highest quartile of TyG (44). A retrospective cohort study from South Korea indicated that the TyG is a significant predictor of CVD and mortality in diabetic patients (45). Among young diabetes patients (aged 20–65 years), a TyG index exceeding the threshold (all-cause mortality: TyG ≥ 9.18; CVD mortality: TyG ≥ 9.16) was significantly positively correlated with both all-cause mortality and CVD mortality (46). Research by Zheng et al. indicated that elevated TyG and TyG-BMI levels were significantly associated with CVD risk in diabetic patients (47). In patients with AMI, the TyG index was also an effective biomarker for risk stratification in diabetic patients (48). These findings were consistent with our conclusions. However, a key discovery of this study was that TyG-BMI was not only significantly associated with MACEs risk in young diabetic patients but also correlated with MACEs risk in young prediabetic individuals. Compared to the TyG and TG/HDL-C, the TyG-BMI metric may better reflect MACEs risk across different states of glucose metabolism. This study further identified that among individuals with elevated hsCRP levels, TyG-BMI demonstrated a strong association with MACEs risk, suggesting that when TyG-BMI and heightened inflammatory status act in concert, the risk of MACEs may be substantially elevated.

Mechanistically, TyG-BMI, as a composite indicator, simultaneously captures three critical risk signals: dyslipidemia, impaired glucose metabolism, and obesity severity. We hypothesize that the strong association between TyG-BMI and MACE may be mediated through the following key mechanisms:

First, TyG-BMI correlates with systemic chronic inflammation and adipose tissue dysfunction. Obesity represents abnormal adipose tissue expansion, particularly visceral fat accumulation, leading to adipocyte hypoxia and mitochondrial dysfunction. This triggers endoplasmic reticulum stress and local inflammation (49–51). Dysfunctional adipose tissue massively secretes pro-inflammatory cytokines such as tumor necrosis factor-α and interleukin-6, while reducing secretion of adiponectin, which possesses anti-inflammatory and insulin-sensitizing effects (52–54). This results in a persistent, low-grade systemic chronic inflammatory state. Our findings provide compelling support: within the subgroup of patients with elevated hsCRP levels, the association between high TyG-BMI and MACE risk was particularly pronounced. This strongly suggests a synergistic amplification effect between metabolic dysfunction, as represented by TyG-BMI, and inflammatory pathways. Elevated TyG-BMI likely serves as a surrogate marker for adipose tissue dysfunction, exacerbating plaque instability and ultimately precipitating adverse events by driving chronic inflammation.

Second, TyG-BMI is associated with endothelial dysfunction and IR. IR and its associated lipotoxicity are core characteristics of elevated TyG-BMI. Elevated circulating inflammatory mediators, free fatty acids, and triglycerides directly impair insulin signaling pathways. Specifically, by inhibiting tyrosine phosphorylation of insulin receptor substrates (IRS) and activating serine kinase pathways (e.g., IKKβ/NF-κB and JNK pathways), thereby further disrupting insulin signaling and severely impairing downstream insulin signaling in the PI3K/Akt pathway. This ultimately prevents glucose transporter 4 (GLUT4) from translocating to the cell membrane, manifesting as systemic IR (53–57). Inflammatory mediators and excess FFAs jointly promote excessive reactive oxygen species (ROS) production, triggering oxidative stress; simultaneously, they downregulate endothelial nitric oxide synthase (eNOS) activity (58). This reduces the bioavailability of nitric oxide (NO), which possesses vasoprotective effects, and upregulates adhesion molecules such as vascular cell adhesion molecule-1 (VCAM-1), collectively leading to endothelial dysfunction (59–62). This promotes leukocyte adhesion, increased vascular permeability, and impaired vasodilation, thereby accelerating the initiation and progression of atherosclerosis. It disrupts coronary microcirculation, promotes restenosis and plaque rupture, and consequently elevates the risk of MACEs.

Importantly, TyG-BMI demonstrates a synergistic amplification effect and plays a prominent role in specific high-risk populations. TyG-BMI is not a simple sum of its components but produces synergistic effects. The obesity burden represented by BMI exacerbates IR, which in turn stimulates the liver to synthesize more very low-density lipoproteins, leading to hypertriglyceridemia. This mechanism effectively explains why TyG-BMI demonstrates the strongest risk predictive capability (HR: 3.85) among PMI patients with concomitant diabetes. Obesity-related chronic inflammation synergizes with diabetes-intrinsic metabolic disorders to further amplify damage to the vascular endothelium. In summary, the robust predictive value of the TyG-BMI stems from its ability to comprehensively reflect chronic inflammation driven by adipose tissue dysfunction, endothelial injury caused by IR, and lipid-glucose metabolic disorders.

TyG-BMI’s outstanding predictive performance highlights its potential clinical value in secondary prevention risk stratification. First, as a simple and cost-effective tool derived from routine testing, it helps identify individuals with higher revascularization needs or greater susceptibility to readmission due to unstable angina, thereby requiring closer monitoring and more aggressive risk factor management. Our findings suggest that TyG-BMI could serve as an effective tool for identifying PMI patients who might derive the greatest benefit from intensified medical nutrition therapy (e.g., low-carbohydrate diets, weight management), thereby facilitating more precise nutritional support. Furthermore, an increase in TyG-BMI during follow-up may indicate worsening insulin resistance, prompting clinicians to pay closer attention to assessing ischemic symptoms and promptly intensifying or adjusting known pharmacological therapies that improve metabolic markers. Incorporating TyG-BMI into post-MI management protocols enables more personalized and dynamic treatment strategies.

## 5 Strengths and limitations

This study possesses several significant advantages. First, its prospective cohort design and methodological rigor: As the first prospective study to systematically evaluate and compare the predictive efficacy of three readily available IR indices (TyG, TyG-BMI, and TG/HDL-C) in patients with PMI, we employed restrictive cubic spline analysis to elucidate dose-response relationships and rigorously evaluated incremental predictive value using Harrell’s C-index, NRI, and IDI, thereby multidimensionally deciphering the clinical significance of each biomarker. Second, clear clinical translational value: The TyG-BMI index, derived from routine laboratory parameters and BMI, is simple to calculate and cost-effective. Its predictive advantage is particularly pronounced in patients with diabetes and individuals with elevated hsCRP. This provides direct justification for incorporating it into existing risk assessment systems to identify high-risk patients requiring enhanced monitoring and intervention (especially nutritional management).

Several limitations existed in this study. First, data collection was conducted at a single center, necessitating large-scale, multicenter studies to further evaluate the value of these indicators. Second, despite adjusting for confounding factors, unaccounted variables such as dietary patterns and exercise frequency may introduce bias. And the study employed left atrial diameter (LA) rather than the standard left atrial volume index (LAVI). Third, while BMI was used as an obesity indicator, it does not reflect fat distribution. Fourth, the proportion of female participants in our cohort was relatively low (11.4%). This sample characteristic significantly limited the generalizability of the study findings to female patients with PMI. Based on the findings and limitations of this study, we propose the following directions for future research: First, the predictive value of TyG-BMI for PMI requires external validation in multicenter, large-scale, gender-balanced, prospective cohorts, with particular attention to its applicability across different age groups, genders, and ethnic groups. Second, integrating TyG-BMI with lifestyle data, more precise body composition measurements (such as skeletal muscle mass, visceral fat content, and waist-to-hip ratio), and imaging data (such as LAVI) holds promise for developing more robust risk prediction models. Finally, design and implement randomized controlled trials based on TyG-BMI risk scores. Such trials could randomly assign patients with high TyG-BMI scores to either standardized treatment groups or personalized nutritional intervention groups to directly validate whether TyG-BMI-guided intervention strategies effectively improve clinical outcomes in PMI patients.

## 6 Conclusion

This prospective study identified that elevated levels of both TyG and TyG-BMI are associated with increased risk of MACEs in patients with PMI. TyG-BMI demonstrated significantly superior predictive performance compared to TyG and TG/HDL-C, particularly in patients with concomitant diabetes and high levels of hsCRP. TyG-BMI serves as an independent predictor and reliable biomarker for the precise management of the prognosis in PMI patients.

## Data Availability

The raw data supporting the conclusions of this article will be made available by the authors, without undue reservation.
