# Supplementary material for: The association between insulin resistance indices and the occurrence of major adverse cardiovascular events in patients with premature myocardial infarction: a prospective cohort study
Source: Front Nutr. 2026 Feb 19;13:1724362. doi: 10.3389/fnut.2026.1724362 (PMC12960170; doi:10.3389/fnut.2026.1724362)
Supplement: Supplementary file 1 [file Table_1.docx]

Supplementary Material

**Supplementary Table 1.** Baseline characteristics of study population stratified by MACEs

| **Characteristics** | **Overall** | **Non-MACE(n=1477)** | **MACE(n=211)** | ***P* value** |
| --- | --- | --- | --- | --- |
| Age, years | 42.0 (37.0, 44.0) | 41.0 (37.0, 44.0) | 42.0 (37.0, 45.0) | 0.195 |
| Male, n(%) | 1496 (88.6) | 1310 (88.7) | 186 (88.2) | 0.908 |
| BMI, kg/m^2^ | 26.1 (24.2, 28.4) | 26.0 (24.2, 28.1) | 27.0 (24.8, 29.3) | 0.001 |
| HRate, bpm | 76.0 (67.8, 86.0) | 76.0 (67.0, 86.0) | 76.0 (69.0, 86.0) | 0.209 |
| SBP, mmHg | 131.0 (120.0, 144.0) | 131.0 (120.0, 144.0) | 130.0 (119.0, 145.0) | 0.880 |
| DBP, mmHg | 80.0 (70.0, 90.0) | 80.0 (70.0, 90.0) | 80.0 (70.0, 90.0) | 0.986 |
| Medical history, n(%) |  |  |  |  |
| Smoking | 1054 (62.4) | 915 (61.9) | 139 (65.9) | 0.305 |
| Drinking | 549 (32.5) | 478 (32.4) | 71 (33.6) | 0.768 |
| Hypertension | 810 (48.0) | 704 (47.7) | 106 (50.2) | 0.531 |
| Diabetes | 341 (20.2) | 284 (19.2) | 57 (27.0) | 0.011 |
| Prior MI | 86 (5.1) | 66 (4.5) | 20 (9.5) | 0.003 |
| Hyperlipemia | 360 (21.3) | 321 ( 21.7) | 39 ( 18.5) | 0.323 |
| Angina | 205 (12.1) | 174 (11.8) | 31 (14.7) | 0.272 |
| Family CAD | 154 (9.1) | 134 (9.1) | 20 (9.5) | 0.949 |
| AF | 21 (1.2) | 21 (1.4) | 0 (0.0) | 0.158 |
| Laboratory data |  |  |  |  |
| hsCRP, mg/L | 5.0 (2.3, 9.6) | 5.0 (2.2, 9.6) | 5.5 (2.9, 9.8) | 0.107 |
| Cr, umol/L | 74.0 (64.0, 84.0) | 73.8 (64.0, 84.0) | 76.0 (64.0, 89.0) | 0.053 |
| eGFR, mL/min/1.73 m² | 111.5 (101.2, 117.2) | 111.6 (101.8, 117.2) | 110.2 (93.8, 116.7) | 0.039 |
| UA, umol/L | 360.0 (295.0, 431.0) | 360.0 (295.0, 429.0) | 363.0 (296.5, 445.0) | 0.449 |
| ALT, U/L | 43.4 (28.5, 68.8) | 43.4 (28.0, 68.7) | 42.2 (30.9, 73.0) | 0.560 |
| AST, U/L | 111.6 (47.2, 221.0) | 108.9 (46.4, 220.9) | 121.5 (52.8, 223.4) | 0.354 |
| FPG, mmol/L | 5.8 (5.1, 7.6) | 5.8 (5.1, 7.5) | 6.5 (5.2, 9.1) | <0.001 |
| HbA1c, % | 5.8 (5.4, 7.0) | 5.8 (5.4, 6.9) | 6.0 (5.5, 8.0) | 0.001 |
| TC, mmol/L | 4.8 (4.1, 5.5) | 4.8 (4.1, 5.5) | 4.9 (4.2, 5.7) | 0.207 |
| TG, mmol/L | 2.1 (1.5, 3.1) | 2.1 (1.5, 3.2) | 2.1 (1.5, 3.1) | 0.544 |
| HDL-C, mmol/L | 0.9 (0.8, 1.1) | 0.9 (0.8, 1.1) | 0.9 (0.8, 1.1) | 0.379 |
| LDL-C, mmol/L | 3.1 (2.5, 3.8) | 3.1 (2.4, 3.8) | 3.2 (2.5, 4.0) | 0.184 |
| VLDL-C, mmol/L | 0.6 (0.4, 0.9) | 0.6 (0.4, 0.9) | 0.6 (0.4, 0.9) | 0.925 |
| ApoA1, g/L | 1.1 (1.0, 1.3) | 1.1 (1.0, 1.3) | 1.1 (1.0, 1.3) | 0.301 |
| ApoB, g/L | 1.1 (0.9, 1.4) | 1.1 (0.9, 1.3) | 1.2 (1.0, 1.4) | 0.059 |
| NT-proBNP, pg/mL | 155.6 (30.1, 479.4) | 148.7 (28.7, 449.5) | 240.7 (37.4, 786.5) | 0.003 |
| TyG | 9.2 (8.8, 9.8) | 9.2 (8.8, 9.8) | 9.3 (8.9, 10.0) | 0.014 |
| TyG-BMI | 243.0 (219.3, 272.2) | 241.2 (218.3, 271.3) | 251.1 (227.9, 280.5) | <0.001 |
| TG/HDL-C | 5.1 (3.4, 8.0) | 5.1 (3.3, 7.9) | 5.3 (3.4, 8.1) | 0.448 |
| Echocardiography | | | | |
| LA, mm | 36.0 (34.0, 39.0) | 36.0 (34.0, 39.0) | 37.0 (34.0, 39.0) | 0.033 |
| LV, mm | 51.0 (49.0, 55.0) | 51.0 (49.0, 55.0) | 52.0 (49.0, 56.0) | 0.138 |
| LVEF, % | 52.0 (46.0, 57.0) | 52.0 (46.0, 57.0) | 50.0 (42.0, 56.0) | <0.001 |
| SMART risk score, % | 78.0 (66.0, 88.0) | 78.0 (66.0, 88.0) | 81.0 (67.0, 89.0) | 0.076 |
| Type of AMI | | | | |
| STEMI | 1333 (79.0) | 1172 (79.4) | 161 (76.3) | 0.355 |
| Number of diseased vessels, % | | | | |
| No obvious lesion | 45 (2.7) | 39 (2.6) | 6 (2.8) | 0.027 |
| 1-vessel disease | 648 (38.4) | 584 (39.5) | 64 (30.3) |  |
| 2-vessel disease | 475 (28.1) | 416 (28.2) | 59 (28.0) |  |
| 3-vessel disease | 520 (30.8) | 438 (29.7) | 82 (38.9) |  |
| Location of target lesions, % | | | | |
| RCA | 998 (59.1) | 870 (58.9) | 128 (60.7) | 0.681 |
| LAD | 1292 (76.5) | 1117 (75.6) | 175 (82.9) | 0.024 |
| LCX | 837 (49.6) | 721 (48.8) | 116 (55.0) | 0.109 |
| LM | 73 (4.3) | 62 (4.2) | 11 (5.2) | 0.619 |
| Number of stents, % |  |  |  |  |
| 0 | 374 (22.2) | 318 (21.5) | 56 (26.5) | 0.276 |
| 1 | 957 (56.7) | 844 (57.1) | 113 (53.6) |  |
| 2 | 273 (16.2) | 238 (16.1) | 35 (16.6) |  |
| ≥3 | 84 (5.0) | 77 (5.2) | 7 (3.3) |  |
| Treatment |  |  |  |  |
| Aspirin, % | 1683 (99.7) | 1473 (99.7) | 210 (99.5) | 1.000 |
| Statin, % | 1654 (98.0) | 1449 (98.1) | 205 (97.2) | 0.513 |
| P2Y12 receptor antagonist, % | 1684 (99.8) | 1473 (99.7) | 211 (100.0) | 1.000 |
| Beta blocker, % | 1321 (78.3) | 1150 (77.9) | 171 (81.0) | 0.338 |
| ACEI/ARB, % | 1137 (67.4) | 985 (66.7) | 152 (72.0) | 0.141 |

Notes: MI, myocardial infarction; CAD, coronary artery disease; STEMI, ST-segment elevation myocardial infarction; NSTEMI, non-ST-segment elevation myocardial infarction; HRate, heart rate; hs-CRP, high-sensitivity c-reactive protein; HbA1c, glycosylated hemoglobin; FPG, fasting plasma glucose; TC, total cholesterol; TG, triglycerides; HDL-C, high-density lipoprotein cholesterol; LDL-C, low-density lipoprotein cholesterol; VLDL-C, very-low-density lipoprotein cholesterol; ApoA1, apolipoprotein A1; ApoB, apolipoprotein B; TyG-BMI, triglyceride glucose body mass index; Cr, creatinine; UA, uric acid; ALT, alanine aminotransferase; AST, aspartate aminotransferase; NT-proBNP, N-terminal pro-B-type natriuretic peptide.

**Supplementary Table 2.** Multicollinearity Analysis

| **Variable** | **GVIF** | **Df** | **GVIF^(1/(2*Df))** |
| --- | --- | --- | --- |
| TyG-BMI | 1.29380 | 3 | 1.04387 |
| Age | 1.44132 | 1 | 1.20055 |
| Gender | 1.50918 | 1 | 1.22849 |
| Smoking | 1.33174 | 1 | 1.15401 |
| Drinking | 1.24382 | 1 | 1.11527 |
| Hypertension | 1.18328 | 1 | 1.08779 |
| Diabetes | 1.13238 | 1 | 1.06413 |
| Hyperlipemia | 1.16311 | 1 | 1.07848 |
| Prior MI | 1.14715 | 1 | 1.07105 |
| LVEF | 1.36561 | 1 | 1.16859 |
| LA | 1.26387 | 1 | 1.12422 |
| NT-proBNP | 1.29472 | 1 | 1.13786 |
| hsCRP | 1.15063 | 1 | 1.07268 |
| ApoB | 1.19320 | 1 | 1.09234 |
| UA | 1.64573 | 1 | 1.28286 |
| eGFR | 1.62176 | 1 | 1.27348 |
| SBP | 2.50793 | 1 | 1.58364 |
| DBP | 2.41685 | 1 | 1.55462 |

**Supplementary Table 3.** Distribution of variables with missing data

| Variable | Number | Missing proportion |
| --- | --- | --- |
| LVEF | 62 | 3.67% |
| LA | 65 | 3.85% |
| NT-proBNP | 138 | 8.18% |
| hsCRP | 73 | 4.32% |
| ApoB | 82 | 4.86% |
| UA | 10 | 0.59% |
| Cr | 10 | 0.59% |

**Supplementary Table 4.** Associations of TyG-BMI with incidences of different outcomes

| Model | HR (95%CI) | | | |
| --- | --- | --- | --- | --- |
|  | Q1 | Q2 | Q3 | Q4 |
| **Cardiac death, n(%)** | 4 (0.9) | 5 (1.2) | 5 (1.2) | 5 (1.2) |
| Model1 | Reference | 1.39 (0.39-5.22) | 1.52 (0.40-5.75) | 1.76 (0.46-6.69) |
| Model2 | Reference | 1.40 (0.37-5.26) | 1.44 (0.38-5.50) | 1.56 (0.41-6.05) |
| Model3 | Reference | 2.73 (0.6-12.49) | 2.25 (0.5-10.19) | 2.36 (0.52-10.76) |
| **Unstable angina, n(%)** | 7 (1.7) | 9 (2.1) | 18 (4.3) | 19 (4.5) |
| Model1 | Reference | 1.45 (0.54-3.91) | 3.39 (1.41-8.17)** | 4.74 (1.96-11.43)*** |
| Model2 | Reference | 1.50 (0.56-4.05) | 3.84 (1.59-9.32)** | 4.75 (1.95-11.53)*** |
| Model3 | Reference | 1.36 (0.50-3.67) | 3.19 (1.28-7.93)* | 3.15 (1.24-8.03)* |
| **Non-fatal MI, n(%)** | 5 (1.2) | 5 (1.2) | 7 (1.7) | 5 (1.2) |
| Model1 | Reference | 1.16 (0.33-4.03) | 1.91 (0.60-6.06) | 1.78 (0.50-6.37) |
| Model2 | Reference | 0.89 (0.25-3.21) | 1.41 (0.42-4.73) | 1.58 (0.42-5.92) |
| Model3 | Reference | 0.89 (0.24-3.26) | 1.61 (0.45-5.70) | 1.58 (0.39-6.34) |
| **HF, n(%)** | 9 (2.1) | 10 (2.4) | 8 (1.9) | 9 (2.1) |
| Model1 | Reference | 1.21 (0.49-2.98) | 1.13 (0.43-2.94) | 1.58 (0.62-4.06) |
| Model2 | Reference | 1.14 (0.46-2.84) | 1.04 (0.39-2.76) | 1.31 (0.50-3.41) |
| Model3 | Reference | 1.38 (0.52-3.61) | 0.88 (0.31-2.46) | 1.29 (0.44-3.79) |
| **Revascularization, n(%)** | 10 (2.4) | 18 (4.3) | 23 (5.5) | 23 (5.5) |
| Model1 | Reference | 1.91 (0.88-4.14) | 2.71 (1.28-5.73)** | 3.36 (1.59-7.12)** |
| Model2 | Reference | 1.86 (0.86-4.05) | 2.69 (1.26-5.74)* | 3.34 (1.56-7.14)** |
| Model3 | Reference | 1.88 (0.86-4.10) | 2.81 (1.30-6.07)** | 3.54 (1.60-7.84)** |

Model 1, adjusted for age and gender; Model 2, adjusted for age, gender, smoking, drinking, medical history of hypertension, diabetes, hyperlipemia, and Prior MI; Model 3, adjusted for variables included in Model 2 and LVEF, LA, NT-proBNP, SBP, DBP, hsCRP, ApoB, UA, eGFR. Unstable angina: rehospitalization for unstable angina. HF: rehospitalization for severe heart failure. **p*<0.05, ***p*<0.01, ****p*<0.001.


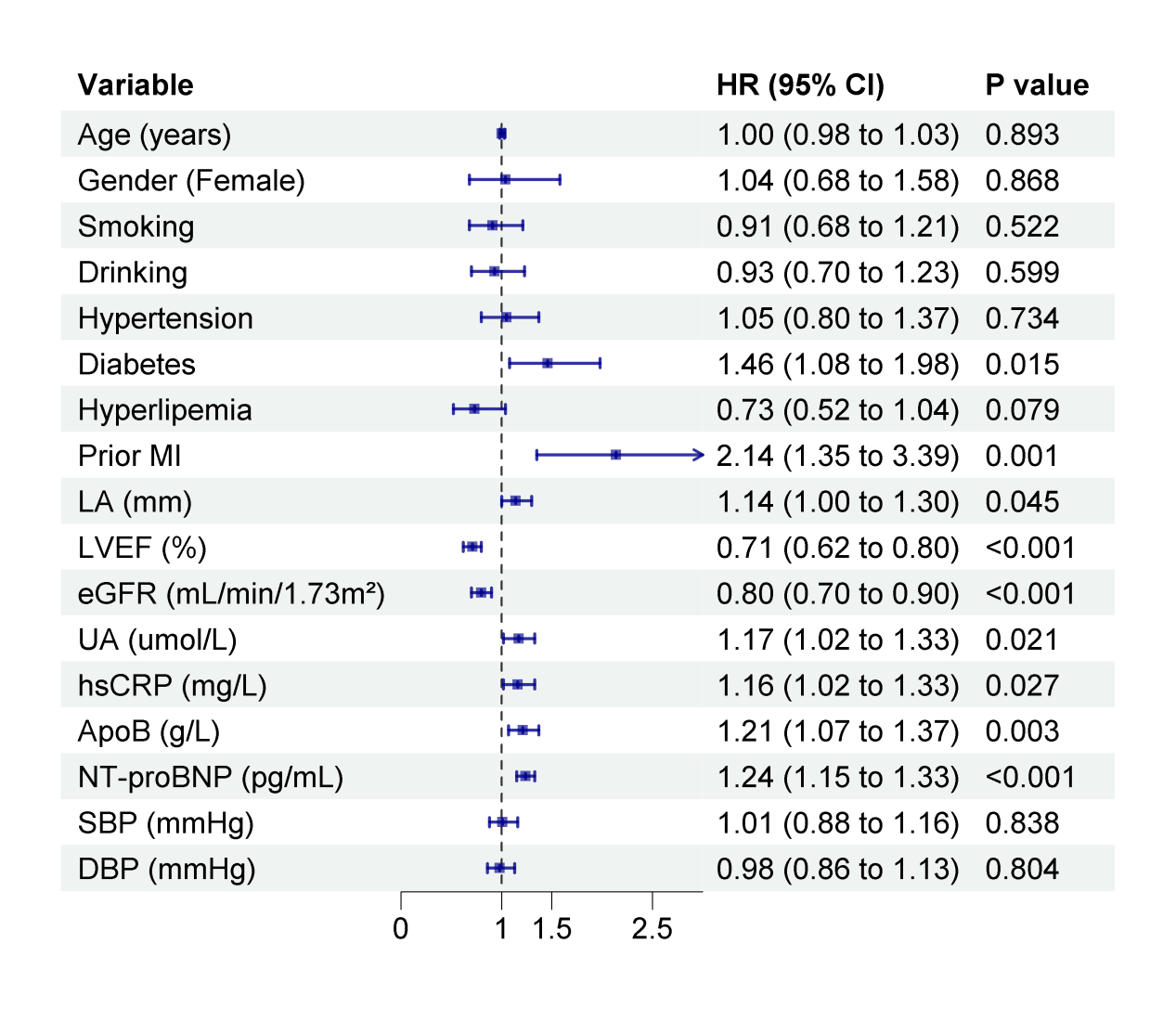


**Supplementary Figure 1.** Univariate analysis of covariates in the adjusted model.


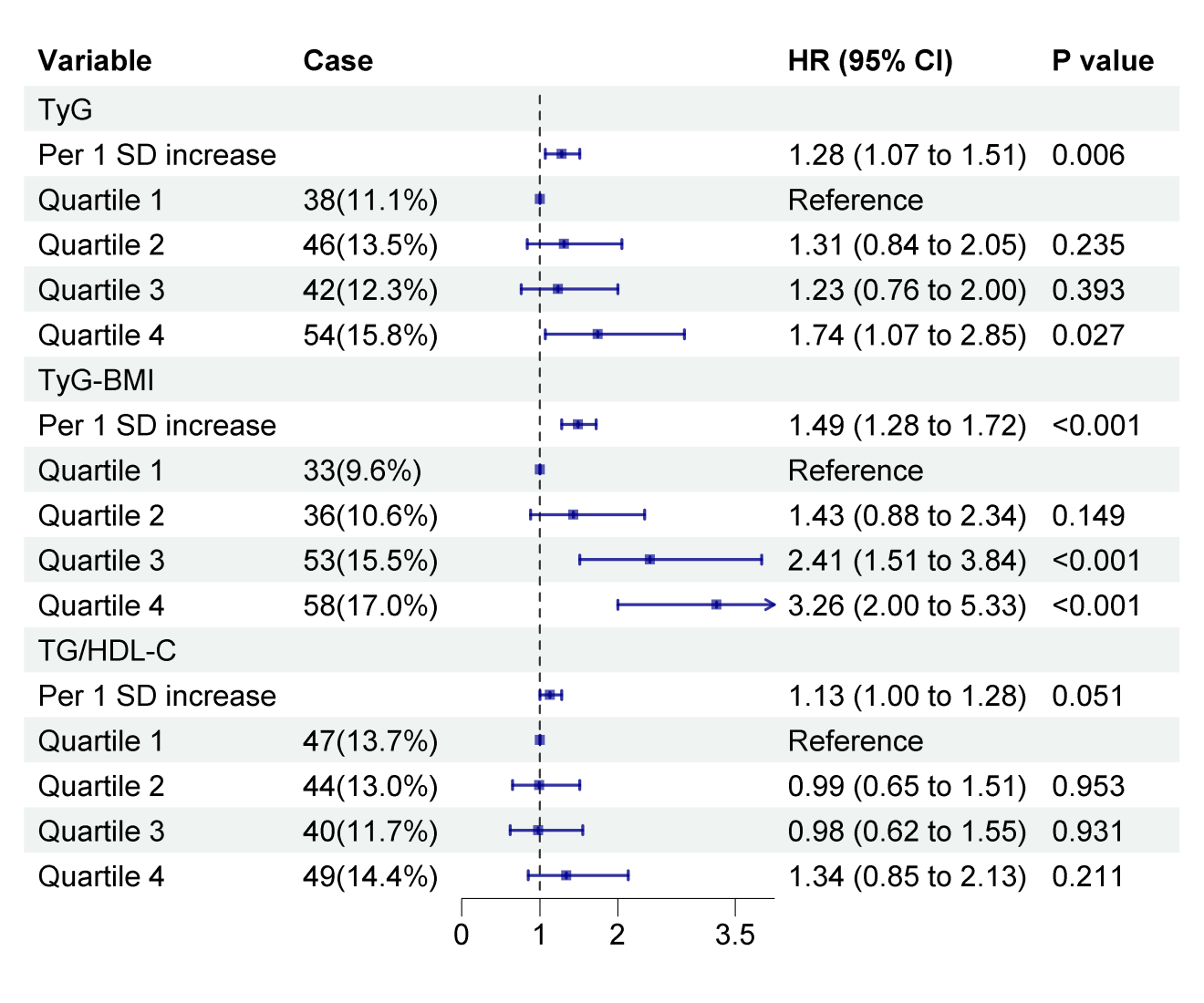


**Supplementary Figure 2.** Sensitivity analyses of the associations between IR indices and MACEs in participants with PMI after excluding missing data of covariates.

Model was adjusted for gender, age, smoking, drinking, and history of hypertension, diabetes, hyperlipemia, Prior MI, and LVEF, LA, NT-proBNP, SBP, DBP, hsCRP, ApoB, UA, eGFR.

TyG：Q1, TyG≤8.78. Q2, 8.78<TyG≤9.19. Q3, 9.19<TyG≤9.77. Q4, TyG>9.77. TyG-BMI: Q1, TyG-BMI≤217.88. Q2, 217.88<TyG-BMI≤241.73. Q3, 241.73<TyG-BMI≤271.08. Q4, TyG-BMI>271.08. TG/HDL-C：Q1. TG/HDL-C≤3.30. Q2, 3.30<TG/HDL-C≤5.06. Q3, 5.06<TG/HDL-C≤7.87. Q4, TG/HDL-C>7.87.
